# Accumulation of nucleotide substitutions occurring during experimental transmission of foot-and-mouth disease virus

**DOI:** 10.1099/vir.0.046029-0

**Published:** 2013-01

**Authors:** Nicholas Juleff, Begoña Valdazo-González, Jemma Wadsworth, Caroline F. Wright, Bryan Charleston, David J. Paton, Donald P. King, Nick J. Knowles

**Affiliations:** The Pirbright Institute, Ash Road, Pirbright, Woking, Surrey GU24 0NF, UK

## Abstract

Analysis of full-genome sequences was previously used to trace the origin and transmission pathways of foot-and-mouth disease virus (FMDV) outbreaks in the UK in 2001 and 2007. Interpretation of these data was sometimes at variance with conventional epidemiological tracing, and was also used to predict the presence of undisclosed infected premises that were later discovered during serological surveillance. Here we report the genome changes associated with sequential passage of a highly BHK-21-cell-adapted (heparan sulphate-binding) strain of FMDV arising from two independent transmission chains in cattle. *In vivo* virus replication rapidly selected for a wild-type variant with an amino acid substitution at VP3^56^. Full-genome sequence analysis clearly demonstrated sequence divergence during parallel passage. The genetic diversity generated over the course of infection and the rate at which these changes became fixed and were transmitted between cattle occurred at a rate sufficient to enable reliable tracing of transmission pathways at the level of the individual animal. However, tracing of transmission pathways was only clear when sequences from epithelial lesions were compared. Sequences derived from oesophageal–pharyngeal scrapings were problematic to interpret, with a varying number of ambiguities suggestive of a more diverse virus population. These findings will help to correctly interpret full-genome sequence analyses to resolve transmission pathways within future FMDV epidemics.

## Introduction

Foot-and-mouth disease (FMD) is a highly contagious, acute viral disease of cloven-hoofed animals, characterized by fever, loss of appetite, depression, lameness and the appearance of vesicles on the feet and in, or around, the mouth. Recovery generally occurs within 8–15 days; however, ruminants can carry FMD virus (FMDV) in the oropharynx for years following the resolution of acute infection (Alexandersen *et al.*, 2002). The virus can spread extremely rapidly (partly as a consequence of the small amount of virus that can initiate infection, the large amount of virus excreted by affected animals and the multiple routes of infection) by direct or indirect contact with infected animals or their products or by long-distance airborne transmission (Alexandersen *et al.*, 2003). FMDV has the potential to cause enormous economic losses and is the single most important constraint to international trade in livestock and animal products (Perry & Rich, 2007).

FMDV belongs to the genus *Aphthovirus* (family *Picornaviridae*). The FMDV virion consists of an icosahedral shell composed of 60 copies each of the four structural proteins, VP1–VP4 (Rueckert & Wimmer, 1984). The capsid proteins surround a positive-sense ssRNA genome approximately 8.3 kb in size that replicates via a negative-strand intermediate (Belsham, 2005). FMDV, in common with other RNA viruses, displays very high mutation rates during replication, corresponding to the poor fidelity of the RNA polymerases (Domingo *et al.*, 2001). The mutation rate, together with other factors, for example the genomic architecture, replication speed and recombination, determines the rate at which a virus population evolves (Duffy *et al.*, 2008). Commonly cited mutation rates for RNA viruses lie in the range 10^−3^–10^−5^ mutations nt^−1^ per genomic replication (Drake & Holland, 1999). FMDV RNA polymerase is no exception, with current estimates suggesting that, on average, one nucelotide sequence change occurs during each round of virus replication (Haydon *et al.*, 2001). In the field, the high mutation rate, fast replication cycle and large size of the affected population results in the rapid evolution of FMDV (Domingo *et al.*, 2003). Molecular epidemiology studies to monitor the evolution of the relatively short nucleotide sequences encoding the capsid VP1 protein provide valuable insight into the emergence of new strains and serotypes worldwide (Ayelet *et al.*, 2009; Valarcher *et al.*, 2009). However, full-genome sequencing of FMDV provides greater resolution when reconstructing transmission pathways, as demonstrated in the UK epidemic in 2001 and in 2007 (Cottam *et al.*, 2006, 2008b). These reports are based on consensus genomes sequenced directly from epithelial lesions, blood or oesophageal–pharyngeal scrapings (probang samples) collected from individual animals on infected premises (IPs). The 2001 post-outbreak investigation retrospectively resolved inter-farm transmission of FMDV (Cottam *et al.*, 2006, 2008a; König *et al.*, 2009). In 2007, full-genome sequencing was used in ‘real time’ to identify the initial outbreak virus and to connect the first and second temporal clusters of IPs (Cottam *et al.*, 2008b). These data were invaluable for informing control policies, they predicted the existence of undetected intermediate premises that were subsequently identified, and demonstrated that full-genome sequence analysis has the required resolution for inter-farm transmission tracing (Cottam *et al.*, 2008b; Schley *et al.*, 2008). The initial outbreak virus was identified as O_1_/BFS 1860/UK/67, a strain propagated extensively in cell culture for vaccine production (Cottam *et al.*, 2008b). Field isolates of FMDV use integrin heterodimeric (α/β) glycoproteins as cellular receptors through an interaction mediated by the Arg-Gly-Asp (RGD) motif within VP1 (Jackson *et al.*, 2000). Serial passage of FMDV in cell culture may select for viruses that also bind to heparan sulphate through acquisition of positively charged amino acid residues at VP2^134^ and/or VP3^56^ (Fry *et al.*, 1999; Jackson *et al.*, 1996; Sa-Carvalho *et al.*, 1997). An additional change from a negatively charged amino acid at VP3^60^ to a neutral residue often occurs; however, this change is not considered essential for heparan sulphate binding (Sa-Carvalho *et al.*, 1997). These heparan sulphate-binding phenotypes are considered to be attenuated in host species (Sa-Carvalho *et al.*, 1997). Herein we report the genome changes associated with reversion to *in vivo* replication for a highly cell-adapted strain of FMDV during sequential, parallel passage in a controlled study in cattle. Despite having undergone extensive passage in cell culture, *in vivo* virus replication in the natural host rapidly selected for a wild-type variant with an amino acid substitution at VP3^56^. We demonstrate that FMDV transmission can be reliably traced at the level of the individual animal, with clear divergence of sequences during sequential, parallel passage.

## Results

### FMDV transmission and disease presentation

**Fig. 1.**
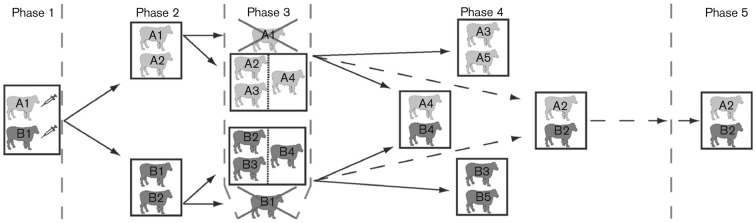
FMDV infection and calf-to-calf transmission. Phase 1: two calves (A1 and B1) were challenged by subepidermolingual injection and housed in the same box for 24 h. Phase 2: each inoculated calf was moved into a separate box to challenge a naïve calf (A2 or B2) by direct-contact exposure. Phase 3: after 5 days of direct-contact exposure, vesicular lesions were detected in the oral cavity, on the tongue, snout or mouth area of the contact-exposed calves. Inoculated calves A1 and B1 were removed from the study. Calves A2 and B2 were moved to the portioned boxes, each containing two naïve calves, one for direct-contact challenge (A3 or B3) and a second for indirect-contact challenge (A4 or B4). Phase 4: following a 24 h challenge, the two indirect-contact challenged calves (A4 and B4) were removed from the portioned boxes and housed together for 14 days. The direct-contact challenged calves (A3 and B3) were moved to separate boxes containing a naïve calf (A5 or B5) for an additional direct-contact challenge for 14 days. Calves A2 and B2 were moved into a single box and maintained up to 32 days p.c. (phase 5).

**Fig. 2.**
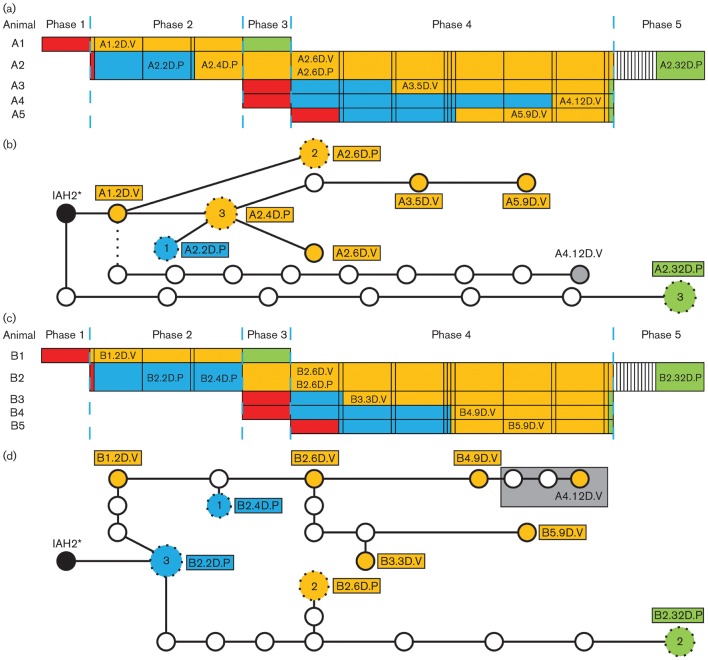
FMD progression and phylogenetic trees. (a, c) Tabular representations of the experiment (Fig. 1), highlighting samples selected for sequencing. Each column of the table under phases 1–5 represents a study day. FMDV infection and disease presentation are colour-coded for each animal: red shading represents initial day of challenge, light-blue shading represents incubation period before clinical FMD, orange shading represents period of clinical FMD and green shading represents day of termination. Samples are coded AX or BX.YD.V or P: AX or BX, animal number for group A or B, respectively; YD, days post-challenge (p.c.); V, vesicular epithelium or vesicular fluid; P, probang sample (oesophageal–pharyngeal scraping sample). (b, d) Phylogenetic trees constructed from the viral sequences derived from the A or B groups, respectively. Samples are colour-coded as described above, with lines between circles representing a single nucleotide change. Samples that contained consensus-sequence ambiguities are shown as larger circles (labelled with the actual number of ambiguities that were present), which are linked to other samples via the closest sequence contained within the population. Sample A4.12D.V is represented by a grey circle and dashed line (from A1.2D.V) in (b), as the transmitted virus originated from animal B4 and is part of the B transmission group (d).

Pyrexia, nasal discharge and large vesicles on the feet and within the oral cavity, secondary to the inoculation sites, were present within 24 h of intradermolingual challenge (Figs 1 and 2). All calf-to-calf challenges resulted in clinical FMD, with epithelial vesicles detected in the oral cavity, tongue and on all four feet of all animals. Vesicular fluid or epithelium from ruptured vesicles was collected immediately upon detection, or within 24 h if detected before calf-to-calf challenge in order to prevent artificial environmental contamination. The samples selected for sequencing (as described in Methods) are shown in Fig. 2.

### Maximum-parsimony analysis and transmission pathways

Three phylogenetic trees were constructed, including: (i) all the sequences determined (not shown); (ii) only the sequences from viruses isolated from animals in the A group (Fig. 2b); and (iii) the sequences from viruses isolated from animals in the B group (Fig. 2d). The transmission events based on sequences isolated from epithelial lesions were clear and unambiguous, with one to four nucleotide changes for each calf-to-calf transmission: IAH2 (a tissue-culture-adapted FMDV with a heparan sulphate-binding phenotype) to A1 (1 nt), A1 to A2 (2 nt), A3 to A5 (1 nt), IAH2 to B1 (4 nt), B1 to B2 (2 nt), B2 to B3 (4 nt) and B2 to B4 (1 nt). However, some events were complicated by apparent back-mutations: A2.6D.V to A3.5D.V (Fig. 2b) and B3.3D.V to B5.9D.V (Fig. 2d). Nevertheless, this may indicate that these particular virus isolates were not part of the direct line of transmission and the apparent back-mutations actually existed in another site in the same animal. The combined tree clearly indicated that the virus infecting calf A4 was derived from calf B4 (three nucelotide differences as opposed to 10 if it had originated from donor calf A2). Calves A4 and B4 (indirect contacts with calves A2 and B2, respectively) were housed together following challenge (Fig. 1) and FMD lesions were recorded for calf B4 3 days before calf A4, providing a window during the period of direct contact for virus transmission.

Sequences derived from oesophageal–pharyngeal scrapings were problematic to interpret, with a varying number of ambiguities in all sequenced samples and frequently diverging from the main branch of transmitted virus (Fig. 2). Interestingly, the viral sequences derived from calf A2 and B2 oesophageal–pharyngeal scrapings at 32 days post-challenge (p.c.) were phylogenetically more closely related to the inoculum than to viral sequences derived from epithelial lesions from both animals at 6 days p.c. (seven to nine changes compared with 10 to 13 changes, respectively). This suggests that virus may evolve independently in different sites in the same animal and this may be more obvious in the oesophageal–pharyngeal area, where the virus can establish infection and persist for long periods.

### Distribution and analysis of evolutionary changes at the consensus level

A total of 49 nucleotide substitutions at 47 sites along the genome were detected in 18 consensus sequences (Table 1). The genetic variation observed between viruses was mainly due to synonymous point substitutions, the majority of which were transitions. The non-synonymous substitutions resulted in 10 amino acid substitutions (Table 2). The transition : transversion ratio for the A transmission chain, excluding sequence A4.12D.V, was 6.67. The ratio for the B transmission chain, which included A4.12D.V, was 3.67, demonstrating a strong bias towards transition substitutions. The 3′ UTR and 3B1 and 3B2 coding regions contained the greatest number of substitutions per site (0.056, 0.029 and 0.028, respectively; Table 1). There were no changes detected in the VP4, 2A or 3B3 coding regions. One to three ambiguities at the consensus level were detected in all of the sequence traces derived from oesophageal–pharyngeal scraping samples. In comparison, no ambiguities were detected in sequence traces derived from epithelial lesions.

**Table 1.** Distribution of evolutionary changes (as shown by nucleotide substitutions in viral proteins) at the consensus level The sequences derived from oesophageal–pharyngeal samples are in bold. These sequences contained ambiguities and frequently diverged from the main branch of the transmitted virus (Fig. 2).

Sequence*	GenBank accession no.	Viral protein/region affected by nucleotide substitution																		
5′ UTR							Leader			VP2			VP3					
0.006†							0.005			0.005			0.009					
		52‡	54	170	258	552	747	811	1104	1158	1181	2066	2377	2434	2771	2772	2773	2932	2972	3037
IAH2	EU448369	C	T	C	G	T	A	A	C	T	A	G	T	C	C	G	C	C	A	T
A1.2D.V	JX570638														T					
**A2.2D.P**	JX570639								**T**						**T**					
**A2.4D.P**	JX570640														**T**					
A2.6D.V	JX570641														T					
**A2.6D.P**	JX570642													**M**	**T**					
**A2.32D.P**	JX570643			**T**							**R**		**Y**			**A**				
A3.5D.V	JX570644	T													T					
A4.12D.V§	JX570645			T		A									T		T			C
A5.9D.V	JX570646	T													T					
B1.2D.V	JX570647			T											T					
**B2.2D.P**	JX570648									**Y**					**Y**					
**B2.4D.P**	JX570649			**T**						**Y**		**A**			**T**					
B2.6D.V	JX570650			T											T		T			
**B2.6D.P**	JX570651				**C**			**G**		**C**					**T**				**G**	
**B2.32D.P**	JX570652			**Y**						**Y**					**T**			**T**	**G**	
B3.3D.V	JX570653		C	T											T		T			
B4.9D.V	JX570654			T											T		T			C
B5.9D.V	JX570655		C	T			C								T		T			

* Samples are coded AX or BX.YD.V or P: AX or BX, animal number for group A or B, respectively; YD, days p.c.; V, vesicular epithelium or vesicular fluid; P, probang sample (oesophageal–pharyngeal scraping sample).

† This row indicates the number of nucleotide substitutions per site.

‡ This row indicates the genome position for each nucleotide substitution.

§ The virus infecting calf A4 (A4.12D.V) was determined to have originated from calf B4 (B4.9D.V); therefore, A4.12D.V is part of the B transmission group.

**Table 1. cont.** Distribution of evolutionary changes (as shown by nucleotide substitutions in viral proteins) at the consensus level, Part II

Sequence*	GenBank accession no.	Viral protein/region affected by nucleotide substitution															
VP1	2B		2C			3A					3B1	3B2	3C		
0.002	0.004		0.003			0.007					0.029	0.028	0.005		
		3433	4192	4216	4639	4799	4888	5398	5452	5750	5842	5872	5932	5950	6082	6175	6410
IAH2	EU448369	T	C	C	A	G	G	G	C	A	A	G	G	A	C	T	G
A1.2D.V	JX570638																
**A2.2D.P**	JX570639																
**A2.4D.P**	JX570640								**Y**								
A2.6D.V	JX570641																
**A2.6D.P**	JX570642																
**A2.32D.P**	JX570643	**C**			**R**								**A**			**C**	
A3.5D.V	JX570644								T						T		
A4.12D.V§	JX570645		T			T											
A5.9D.V	JX570646								T					G	T		
B1.2D.V	JX570647		T														
**B2.2D.P**	JX570648																
**B2.4D.P**	JX570649		**T**			**T**											
B2.6D.V	JX570650		T			T											
**B2.6D.P**	JX570651					**K**	**R**					**C**					
**B2.32D.P**	JX570652							**A**		**G**	**G**	**C**					
B3.3D.V	JX570653		T	T		T											A
B4.9D.V	JX570654		T			T											
B5.9D.V	JX570655		T			T											A

For footnotes, see p. 112.

**Table 1. cont.** Distribution of evolutionary changes (as shown by nucleotide substitutions in viral proteins) at the consensus level, Part III

Sequence*	GenBank accession no.	Viral protein/region affected by nucleotide substitution											
3D							3′ UTR				
0.005							0.056				
		6865	7183	7372	7393	7639	7828	7981	8110	8150	8151	8157	8160
IAH2	EU448369	A	C	C	T	T	C	T	C	C	C	G	C
A1.2D.V	JX570638												
**A2.2D.P**	JX570639			**M**								**A**	
**A2.4D.P**	JX570640									**Y**		**R**	
A2.6D.V	JX570641				C							A	
**A2.6D.P**	JX570642				**Y**								**T**
**A2.32D.P**	JX570643								**T**			**C**	
A3.5D.V	JX570644							C		T			
A4.12D.V§	JX570645	G				C					T		
A5.9D.V	JX570646							C		T			
B1.2D.V	JX570647										T		
**B2.2D.P**	JX570648											**K**	
**B2.4D.P**	JX570649										**T**		
B2.6D.V	JX570650										T		
**B2.6D.P**	JX570651						**T**					**T**	
**B2.32D.P**	JX570652						**T**					**T**	
B3.3D.V	JX570653		T								T		
B4.9D.V	JX570654										T		
B5.9D.V	JX570655		T								T		

For footnotes, see p. 112.

**Table 2.** Distribution of amino acid changes in viral proteins following sequential parallel passage in cattle The sequences derived from oesophageal–pharyngeal samples are in bold. These sequences contained ambiguities and frequently diverged from the main branch of the transmitted virus (Fig. 2).

	Viral protein affected by amino acid substitution									
	Leader			VP2	VP3			2C	3A	3C
	PP338*	PP356	PP364	PP659	PP894		PP961	PP1570	PP1887	PP2107
	P4†	P22	P30	P39	P56		P123	P129	P128	P124
	1104‡	1158	1181	2066	2771	2772	2972	4799	5750	6410
Sequence§										
IAH2	T	L	K	A	R	R	I	A	T	V
A1.2D.V					C					
** A2.2D.P**	I				**C**					
** A2.4D.P**					**C**					
A2.6D.V					C					
** A2.6D.P**					**C**					
** A2.32D.P**			**E/K**||			**H**				
A3.5D.V					C					
A4.12D.V¶					C			S		
A5.9D.V					C					
B1.2D.V					C					
** B2.2D.P**		**P/L**||			**C/R**||					
** B2.4D.P**		**P/L**||		**T**	**C**			**S**		
B2.6D.V					C			S		
** B2.6D.P**		**P**			**C**		**V**	**S/A**||		
** B2.32D.P**		**P/L**||			**C**		**V**		**A**	
B3.3D.V					C			S		I
B4.9D.V					C			S		
B5.9D.V					C			S		I
Charge#	None	None	+ to −	None	+ to N	+ to N	None	None	None	None

* This row indicates the polyprotein position of the amino acid substitution.

† This row indicates the protein position of the amino acid substitution.

‡ This row indicates the genome position for each non-synonymous nucleotide substitution.

§ Samples are coded as explained in Table 1.

|| Ambiguous nucleotide substitutions.

¶ The virus infecting calf A4 (A4.12D.V) was determined to have originated from calf B4 (B4.9D.V); therefore, A4.12D.V is part of the B transmission group.

# Change in charge as a result of non-synonymous nucleotide substitution: None, no change; +, positive; −, negative; N, neutral.

*In vivo* passage of FMDV IAH2 rapidly selected for wild-type variants, with substitution at residue VP3^56^ from Arg to Cys detected at the consensus level in both of the inoculated calves at 2 days p.c. (Tables 1 and 2). This initial selection was conserved at the consensus level through all four parallel calf-to-calf transmissions. However, an oesophageal–pharyngeal scraping from donor animal A2 at 32 days p.c. (A2.32D.P, Fig. 2) contained a variant virus, with a substitution at VP3^56^ from an Arg to a His residue (Tables 1 and 2). No substitutions were detected at positively charged Lys at VP2^134^ or neutral Gly at VP3^60^. Besides the change at nucleotide position 2771 (resulting in a Cys at VP3^56^), only a substitution of C to U at nucleotide position 170, within the 5′ UTR S-fragment, was present in both group A and B (Table 1). In addition, there was one disparate substitution at position 8157 within the 3′ UTR in both groups. All other changes occurred at different nucleotide positions, demonstrating clear sequence divergence between the A and B transmission chains.

## Discussion

Cattle passage of IAH2, a tissue-culture-adapted FMDV with a heparan sulphate-binding phenotype, caused clinical FMD within 24 h and rapidly selected for a wild-type variant. Reversion at the consensus level was detected 2 days p.c. in both inoculated calves by substitution at VP3^56^ from Arg to Cys. This initial selection was conserved at the consensus level through all four calf-to-calf transmissions on two parallel transmission chains. However, an oesophageal–pharyngeal scraping collected from one calf at 32 days p.c. contained a variant with a substitution at VP3^56^ from an Arg to a His residue. No substitutions were detected at VP3^60^, a residue less likely to undergo reversion (Fry *et al.*, 1999; Sa-Carvalho *et al.*, 1997), or at VP2^134^.

Previously reported next-generation sequence analysis of IAH2 revealed only low-frequency Cys and His nucleotide coding substitutions at the VP3^56^ coding region (Table S1, available in JGV Online) (Wright *et al.*, 2011). Single nucleotide polymorphisms (SNPs) at frequencies between 0.17 and 0.06 % at this site are indistinguishable from background, consistent with errors inherent in RNA replication prior to onset of any selective pressure (Wright *et al.*, 2011). No high-frequency SNPs were detected in either the A or the B group transmission chains following parallel *in vivo* passage. The observed substitutions were either random or the result of mutations which rapidly selected against heparan sulphate binding. Clearly, extensive culture passage and subsequent adaptation to bind heparan sulphate did not completely attenuate IAH2 *in vivo*. It was previously demonstrated that a chimeric FMDV O_1_ Campos/A_12_ cDNA clone, with Arg at VP3^56^, was highly attenuated for cattle at lower doses (Sa-Carvalho *et al.*, 1997). However, reinfection with a higher dose resulted in clinical FMD, and recovered virus had reverted back to wild type with an uncharged Cys at VP3^56^ or a negatively charged Glu at VP2^134^ (Sa-Carvalho *et al.*, 1997). A His at VP3^56^ was also detected following *in vivo* passage (Sa-Carvalho *et al.*, 1997). The involvement of Arg at VP3^56^ in viral attenuation for cattle has also been confirmed using O_1_/Campos/Bra/58 cDNA clones (Borca *et al.*, 2012). Similar studies in swine also resulted in delayed onset of FMD, with recovered virus exhibiting reversion to Cys or His at VP3^56^ (Borca *et al.*, 2012). Interestingly, these authors also demonstrated that *in vitro* heating of the viruses at 41 °C caused a pronounced loss of infectivity of VP3^56^ Arg phenotype viruses compared with VP3^56^ His viruses (Borca *et al.*, 2012), which may play a role in selective pressure *in vivo*. Here we show how rapidly a single nucleotide mutation can be selected during *in vivo* passage, and that reversion at VP3^60^ and VP2^134^ are not required for virulence and therefore presumably also for wild-type receptor (integrin) usage.

The UK 2007 FMD outbreak was initiated by escape of O_1_/BFS 1860/UK/67 from the Pirbright complex, leading to two IPs in August and to a second cluster of IPs 6 weeks later and approximately 20 km apart (Schley *et al.*, 2008). Three closely related viruses, IAH1, IAH2 and MAH, used at the Pirbright site were identified as possible source viruses (Cottam *et al.*, 2008b). The heparan sulphate-binding strains MAH and IAH2 are closely related to virus isolated from the first IP (IP1b, six and seven substitutions, respectively). In comparison, IAH1 virus, which lacks residues associated with heparan sulphate binding, differs by 12 substitutions (Cottam *et al.*, 2008b). MAH can be distinguished from IAH2 by one substitution at position 1181 (A to G, a non-synonymous change at amino acid residue 2 of the Leader-b polypeptide) which was also present in all 11 reported field virus sequences (Cottam *et al.*, 2008b). We have shown that IAH2 virus can cause clinical FMD in cattle, with rapid reversion to wild-type (non-heparan sulphate binding) Cys or His at VP3^56^. Sequential passage resulted in accumulation of nucleotide substitutions but did not provide strong selective pressure for reversion at VP3^60^, consistent with all but one of the 2007 field viruses (Cottam *et al.*, 2008b). These data are consistent with evidence that a cell-culture-adapted virus was responsible for the 2007 outbreak. Significantly, we demonstrate clear viral sequence divergence during sequential passage through parallel groups of cattle, even in the face of a selective pressure for receptor usage and *in vivo* replication. Only changes at nt 2771 (VP3^56^) and change C to U at nt 170 were present in both the A and the B transmission chains. The evident divergence of sequences and accumulation of nucleotide substitutions provides support for the interpretation of molecular epidemiological analysis during 2007 that linked the two outbreak clusters, contrary to available field epidemiological evidence (Schley *et al.*, 2008). Cottam *et al.* (2008b) identified 36 nucleotide differences between IAH2 and 11 UK-2007 field isolates. Interestingly, we observed seven of these substitutions following *in vivo* passage of IAH2, although these changes were not present as SNPs above 1 % in the inoculum (Wright *et al.*, 2011). Only a single change at position 170 was common to both the A and the B transmission chains. Taken together, these data may suggest that position 170 was under strong selective pressure during cattle passage. However, the significance of this change is not clear, as it is predicted to convert a G–C base pair of a conserved stem structure into a G–U base pair within the 5′ UTR S-fragment (data not shown).

We have shown that the genetic diversity generated over the course of infection, and the rate at which these changes became fixed and were transmitted between calves, occurred at a rate sufficient to enable reliable tracing of transmission pathways at the level of the individual animal. However, transmission events were only clear and unambiguous when based on sequences derived from epithelial lesions. Sequences from oesophageal–pharyngeal scrapings contained ambiguities, complicating their interpretation, and often diverged from the main branch of transmitted virus. The viral diversity in oesophageal–pharyngeal scrapings may be the result of continuous, low-level virus replication at this site, compared with the transient period of replication, prior to sampling, at sites of vesicular lesions. Alternatively, the diversity observed may simply reflect the lower levels of virus present in these samples, resulting in amplification errors. Further studies, utilizing next-generation genome sequencing technology, are required to understand the intra-host population dynamics of FMDV and to identify the source of viruses for onward transmission to other animals.

Surprisingly, the one to four nucleotide changes that we detected during controlled inter-animal transmission were comparable to the number of changes detected between inter-farm transmissions during the UK FMD epidemic in 2001 and in 2007 (Cottam *et al.*, 2008b). During the 2001 analysis, 23 consensus sequences were recovered directly from epithelial lesions acquired from 21 IPs over approximately 7 months, and the nucleotide changes were estimated to accrue at a mean rate of 1.5 substitutions per farm infection (Cottam *et al.*, 2006). Additional analysis by Cottam and colleagues of 22 consensus sequences from 15 IPs over approximately 3 months indicated a mean of 4.3 substitutions upon farm-to-farm transfer (Cottam *et al.*, 2008a). During the ‘real-time’ analysis in 2007, 11 consensus sequences were recovered directly from epithelial lesions, oesophageal–pharyngeal scrapings or blood samples acquired from 11 IPs over approximately 2 months, and the consensus sequences between farms differed by one to five substitutions (Cottam *et al.*, 2008b). It is conceivable that the small number of nucleotide changes that we detected during controlled inter-calf transmission is in part a consequence of the relatively short period that FMDV-infected cattle are infectious (mean 1.7 days) (Charleston *et al.*, 2011). The short infectious period, combined with the small amount of virus required to initiate infection and multiple routes by which the virus can spread, limits the time available for the virus to accumulate consensus-level substitutions before it is transmitted and establishes infection in the subsequent host. In addition, the rapid replication rate combined with a rapid host immune response reduces the susceptibility to superinfection with closely related strains (Juleff *et al.*, 2009). These inter-animal transmission characteristics of FMDV could also account for the small number of changes detected between IPs in the UK during 2001 and 2007. The small number of substitutions between IPs suggests that the contact network between farms was very high, and there were limited cycles of intra-farm animal-to-animal transmission prior to disease detection and sampling. More in-depth analysis of multiple samples from each IP is required to understand the intra- and inter-farm population dynamics of FMD during the 2007 outbreak. In particular, the possible impact of bottlenecks on transmission dynamics is not clear, for example intra-farm transmission cycles initiated by indirect contact and subsequent low quantities of source virus.

Our results suggest that, following transmission of virus to a subsequent herd, continual intra-farm transmission cycles on the IP or ongoing virus replication in a single host during persistent infection, as detected in oesophageal–pharyngeal scrapings, can generate discrete viral populations that are genetically divergent from the transmitted virus. Analyses of sequences derived from these samples are difficult to interpret and can be misleading, with implications for correct interpretation and resolution of transmission pathways. Therefore an understanding of intra-farm viral dynamics is particularly important when there is a broad spectrum of lesion ages on an IP or when oesophageal–pharyngeal scrapings are the only sample type available for tracing. These data demonstrate that full-genome sequencing can be used to resolve FMDV transmission events at the level of the individual animal and highlight the importance of the sample type and the description of the clinical situation for accurate interpretation of transmission events.

## Methods

### 

#### Inoculum.

The FMDV isolate O_1_/BFS 1860/UK/67 (IAH2) (GenBank accession no. EU448369) used for challenge was originally derived from bovine tongue epithelium received at the Food and Agriculture Organization of the United Nations (FAO) World Reference Laboratory for FMD at Pirbright in 1967 from a farm near Wrexham, England. After extensive passage in baby hamster kidney (BHK-21) cells, it was found to have adapted to utilize heparan sulphate as a cellular receptor (Cottam *et al.*, 2008b).

#### Experimental design.

The experiment was designed for sequential, parallel passage of FMDV through two groups, A and B, each containing five calves (Fig. 1). The experiment consisted of five phases carried out in high-containment large animal isolation boxes at the Pirbright Laboratory. Two calves (A1 and B1) were challenged with FMDV by subepidermolingual injection of 10^5.7^ TCID_50_ (phase 1) as described previously (Henderson, 1952). Each inoculated calf was moved 24 h p.c. into a separate box to challenge a naïve calf (A2 or B2) by direct-contact exposure (phase 2). Vesicular lesions were detected in the oral cavity of calves A2 and B2 5 days p.c. Inoculated calves A1 and B1 were then removed from the study and each contact-exposed calf (A2 and B2) was moved to a separate portioned box to act as a donor to transmit FMDV over 24 h by direct or indirect-contact exposure (phase 3). Each portioned box contained two naïve calves, one for direct-contact challenge (A3 or B3) and a second separated by a 30 cm wide, double-walled wooden-and-mesh partition for indirect-contact challenge (A4 or B4). During the first 6 h, the ventilation in the portioned boxes was turned off, raising the relative humidity to >99 % (Charleston *et al.*, 2011). In addition, a wall-mounted fan was used to direct air from the donors to the indirect-contact calves over the 24 h period. Following challenge, the two indirect-contact challenged calves (A4 and B4) were removed from the portioned boxes and housed together for 14 days (phase 4). The direct-contact challenged calves (A3 and B3) were moved to separate boxes containing a naïve calf (A5 or B5) for an additional direct-contact challenge for 14 days (phase 4). Calves A2 and B2 were moved into a single box and maintained up to 32 days p.c. (phase 5). This was necessary since British Home Office rules precluded the housing of lone animals for extended periods and space was limited. Calves were examined daily during phases 1–4 for signs of clinical FMD. Animal experimentation was approved by the Institute for Animal Health (IAH) Ethical Review Board under the authority of a Home Office project licence in accordance with the Home Office Guidance on the Operation of the Animals (Scientific Procedures) Act of 1986 and associated guidelines.

#### Sample collection and coding for sequencing.

Samples collected and sequenced were coded AX or BX.YD.V or P: AX or BX was the animal number for group A and B, respectively; YD was the number of days p.c.; V was for epithelium or vesicular fluid collected from FMD epithelial lesions and P was for oesophageal–pharyngeal scrapings (probang samples). Vesicular fluid was collected using sterile syringes, diluted to a 10 % solution with M25-phosphate buffer (35 mM Na_2_HPO_4_ . 2H_2_O; 5.7 mM KH_2_PO_4_; pH 7.6; made in-house) and stored at −70 °C until used for RNA extraction. Vesicular epithelium was collected using sterile forceps and tweezers, immersed in cryotubes (Sarstedt) containing 50 % (v/v) glycerol in M25-phosphate buffer and stored at −70 °C until being processed. Approximately 1.5 g vesicular epithelium was ground by using a pestle and mortar and resuspended to a 10 % solution with M25-phosphate buffer. The solution was then centrifuged for 10 min at 3500 ***g*** at room temperature, and the supernatant was removed for RNA extraction. Oesophageal–pharyngeal scrapings collected using probang cups (http://www.wrlfmd.org/find_diagnosis/probang.pdf) were stored at −70 °C until used for RNA extraction.

Vesicular epithelium was collected 2 days p.c. from the hoof of the left pelvic limb of calf A1 (A1.2D.V) and from the hoof of the right pelvic limb of calf B1 (B1.2D.V). Oesophageal–pharyngeal scrapings were collected from calves A2 and B2 on days 2, 4, 6 and 32 p.c. (A2.2D.P, A2.4D.P, A2.6D.P, A2.32D.P and B2.2D.P, B2.4D.P, B2.6D.P, B2.32D.P) and vesicular epithelium was collected 6 days p.c. from the hoof of the left pelvic limb of calves A2 and B2 (A2.6D.V and B2.6D.V). Vesicular epithelium was collected 5 days p.c. from the hoof of the right thoracic limb of calf A3 (A3.5D.V) and vesicular fluid was collected 3 days p.c. from the hoof of the right thoracic limb of calf B3 (B3.3D.V). Vesicular fluid was collected 12 days p.c. or 9 days p.c. from the hoof of the left thoracic limb of calf A4 (A4.12D.V) and B4 (B4.9D.V), respectively. Vesicular fluid was collected 9 days p.c. from the hoof of the right thoracic limb of calf A5 (A5.9D.V) and from the hoof of the left thoracic limb of calf B5 (B5.9D.V).

#### Genome amplification, sequencing and sequence analysis.

Viral RNA was extracted directly from clinical samples using either an RNeasy Mini kit (Qiagen) or TRIzol reagent (Invitrogen) as described previously (Reid *et al.*, 1998). Reverse transcription and DNA amplification were performed as described previously (Cottam *et al.*, 2008b), but with a pre-amplification cDNA purification step using an illustra GFX PCR DNA and Gel Band Purification kit (GE Healthcare UK). Negative controls were used for each step, including control reactions for each of the 24 primer sets used for full-genome amplification. The quantity of viral RNA used was not standardized between the samples that were sequenced.

Amplified PCR products were separated by gel electrophoresis, stained with ethidium bromide and visualized under UV light. After direct purification, cycle sequencing was carried out as described previously using either a Beckman DTCS kit (Beckman Coulter) on a Beckman Coulter CEQ 800 sequencer (Cottam *et al.*, 2008b) or a BigDye Terminator v3.1 Cycle Sequencing kit (Applied Biosystems) on an ABI PRISM 3730 analyser. The SeqMan Pro program from the Lasergene v8.0.2 package (dnastar) was used for assembly and proofreading of sequence trace files. Alignment and manipulation of sequences were performed using BioEdit v7.0.5.3 (Hall, 1999) and DnaSP v4.10.3 (Rozas *et al.*, 2003) software. Maximum-parsimony analysis of the nucleotide sequences was performed using tcs v1.21 (Clement *et al.*, 2000) as described previously (Cottam *et al.*, 2008b). Where ambiguities were present, multiple sequences that contained the different substitutions, but no ambiguities, were constructed; using these sequences, tcs generated complex multi-linked trees. The shortest routes through these trees (which did not involve back-mutations) were chosen to construct the final transmission trees.
